# Neutrophil elastase cleaves epithelial cadherin in acutely injured lung epithelium

**DOI:** 10.1186/s12931-016-0449-x

**Published:** 2016-10-17

**Authors:** Rachel Boxio, Julien Wartelle, Béatrice Nawrocki-Raby, Brice Lagrange, Laurette Malleret, Timothee Hirche, Clifford Taggart, Yves Pacheco, Gilles Devouassoux, Abderrazzaq Bentaher

**Affiliations:** 1 Inflammation and Immunity of the Respiratory Epithelium Group, Faculté de Médecine Lyon Sud, EA 7426, UCBL 1, Inserm U-1111, Pierre Benite - Lyon Sud, France; 2 INSERM UMR-S 903, CHU Maison Blanche, Reims, France; 3 Department of Pulmonary Medicine, German Clinic for Diagnostics (DKD), Wiesbaden, Germany; 4 Centre for Infection and Immunity, Queen’s University Belfast, Belfast, Northern Ireland, UK; 5 CHU Croix-Rousse, Lyon, France

**Keywords:** E-cadherin, Neutrophil elastase, Epithelium disruption, Lung inflammation and injury

## Abstract

**Background:**

In acutely injured lungs, massively recruited polymorphonuclear neutrophils (PMNs) secrete abnormally neutrophil elastase (NE). Active NE creates a localized proteolytic environment where various host molecules are degraded leading to impairment of tissue homeostasis. Among the hallmarks of neutrophil-rich pathologies is a disrupted epithelium characterized by the loss of cell-cell adhesion and integrity. Epithelial-cadherin (E-cad) represents one of the most important intercellular junction proteins. E-cad exhibits various functions including its role in maintenance of tissue integrity. While much interest has focused on the expression and role of E-cad in different physio- and physiopathological states, proteolytic degradation of this structural molecule and ensuing potential consequences on host lung tissue injury are not completely understood.

**Methods:**

NE capacity to cleave E-cad was determined in cell-free and lung epithelial cell culture systems. The impact of such cleavage on epithelial monolayer integrity was then investigated. Using mice deficient in NE in a clinically relevant experimental model of acute pneumonia, we examined whether degraded E-cad is associated with lung inflammation and injury and whether NE contributes to E-cad cleavage. Finally, we checked for the presence of both degraded E-cad and NE in bronchoalveolar lavage samples obtained from patients with exacerbated COPD, a clinical manifestation characterised by a neutrophilic inflammatory response.

**Results:**

We show that NE is capable of degrading E-cad in vitro and in cultured cells. NE-mediated degradation of E-cad was accompanied with loss of epithelial monolayer integrity. Our in vivo findings provide evidence that NE contributes to E-cad cleavage that is concomitant with lung inflammation and injury. Importantly, we observed that the presence of degraded E-cad coincided with the detection of NE in diseased human lungs.

**Conclusions:**

Active NE has the capacity to cleave E-cad and interfere with its cell-cell adhesion function. These data suggest a mechanism by which unchecked NE participates potentially to the pathogenesis of neutrophil-rich lung inflammatory and tissue-destructive diseases.

**Electronic supplementary material:**

The online version of this article (doi:10.1186/s12931-016-0449-x) contains supplementary material, which is available to authorized users.

## Background

To protect itself against various infectious or toxic agents, the lung relies on different mechanisms. Among these latter, the epithelium and resident macrophages are considered as the first lines of lung tissue protection. With respect to the epithelium, this cell lining participates in mounting an appropriate inflammatory response against insulting agents but acts primarily as a physicochemical barrier for efficient defence [1]. Indeed, various structural molecules are known to establish tight epithelial cell-cell adhesion and along with their connection to the internal cytoskeleton provide the lung with an intact and impermeable epithelium [2].

When these “sentinel” lines are breached, neutrophils are called in. The primary purpose of this neutrophilic infiltration is phagocytosis of foreign particles and/or contribution to the resolution of associated inflammation [3]. To this end, two systems categorized as oxygen-dependent and -independent have been described in neutrophils [4]. The non-oxidative system comprises the readily active serine proteinase, neutrophil elastase (NE), among other polypeptides. NE is structurally related to its family members, cathepsin G and proteinase 3, and share the conserved charge-relay triad, His^57^-Asp^102^-Ser^195^, where Ser is the active residue (chymotrypsinogen numbering) [5]. NE, a potent proteolytic enzyme, is stored in an active form at high concentration in primary granules (~4 μg/10^6^ cells) making it a major component of neutrophils [6]. The enzyme is rapidly discharged into the phagolysosome following bacterial uptake by neutrophils. Previously, we and others have shown that NE is required for maximal neutrophil killing of invading pathogens [7–10].

In the setting of overwhelming inflammatory conditions, activated neutrophils secrete abnormally NE in the extracellular space [11]. Active NE creates a localized proteolytic environment where a wide range of host soluble and insoluble molecules could be degraded. As a consequence, NE has been incriminated in the pathogenesis of different acute and chronic tissue-destructive diseases including acute lung injury/acute respiratory distress syndrome (ALI/ARDS), cystic fibrosis (CF) and chronic obstructive pulmonary diseases (COPD) (e.g., emphysema) [12, 13]. Among the common hallmarks of these pathologies is a disrupted epithelium characterized by the loss of its integrity and sloughing of epithelial cells [14]. The likelihood that NE is associated with these diseased situations is supported by at least two lines of evidence. First, NE is potent proteolytic enzyme with a large substrate repertoire comprising extracellular matrix proteins [15]. Second, various human and animal studies reported the presence of free active NE in lung tissues and fluids overwhelming the anti-NE screen [16–20]. Whether NE has the capacity to alter the tight epithelial cell-cell adhesion contributing to lung tissue destruction is not completely addressed particularly in vivo.

E-cad represents one of the most important and ubiquitous adherens junction protein. E-cad, a member of the cadherin superfamily, is a glycoprotein that crosses the membrane only once [21]. The extracellular portion of E-cad consists of a domain of five repeats that are highly homologous to each other and commonly designated as EC1-EC5 (EC1 being the beginning of E-cad N-terminus). Each repeat is comprised of approximately 110 amino acid residues. This extracellular domain ensures homophilic recognition and Ca2 + −dependent cell-cell adhesion. Intracellularly, E-cad cytoplasmic domain is linked to actin filaments through interactions with catenin proteins reinforcing, as mentioned above, the stability of the epithelium [22]. Other functions of E-cad include its role as a signalling molecule and regulator of the epithelium permeability and polarity [23]. The protein is implicated in a number of biological processes including tissue/organ development, morphogenesis, and cytoskeletal organization [23]. Much interest has focused on the expression and role of E-cad in different physio- and physiopathological states. Limited studies have, however, dealt with the proteolytic degradation of this structural molecule by NE and ensuing consequences on host tissue architecture (e.g., contribution to lung tissue injury). In a rat model of pancreatitis, Mayerle J. et al. reported that neutrophil-derived NE, rather than pancreatic elastase, degraded E-Cad [24]. Evans SM and his colleagues observed released E-cad into the BAL fluids in a mouse model challenged with human NE [25]. Work by Downey G. and his colleagues identified a mechanism whereby NE-mediated cleavage of E-cad induced β-catenin signalling, which appears to play a critical role in reepithelialisation of denuded epithelium in a mouse lung inflammation model [26]. The ability of NE to degrade other cell-cell junction proteins prior to E-cad may not be ruled out [27]. Of interest as well, it has been reported that NE mediates degradation of vascular E-cad (VE-cad) that could compromise the endothelial vascular integrity contributing to microvascular injury and increased permeability and interstitial oedema [28].

The goals of these studies were several folds. We sought to determine if NE could cleave E-cad and alter its ability to maintain a stable lung epithelial cell monolayer. Next, using mice deficient in NE in a clinically relevant experimental model of acute pneumonia, we wanted to determine whether degraded E-cad is associated with lung inflammation and injury and whether NE contributes to E-cad cleavage. Finally, in order to determine the potential relevance of our findings to human lung diseases, we examined whether degraded E-cad coincides with the presence of active NE in bronchoalveolar lavage samples obtained from patients with exacerbated COPD, a clinical condition characterized by a neutrophilic inflammatory response.

## Methods

### Reagents

Purified human NE, CG and PR3 and NE corresponding polyclonal rabbit antibody were from Elastin Products Company (Owensville, MO, USA). The purity and activity of each enzyme were confirmed by sodium-dodecyl-sulfate polyacrylamide gel electrophoresis (SDS-PAGE) and spectrophotometrically using specific substrates according to the manufacturer’s recommendations. The chromogenic peptide substrates were N-methoxysuccinyl-Al-Al-Pro-Val-pNA, N-Boc-Ala-ONp, and N-succinyl-Al-Al-Pro-Phe-pNA for NE, PR3 and CG, respectively. Chromogenic peptide substrates were obtained from Elastin Products Company. Recombinant human secretory leukocyte proteinase inhibitor (SLPI) and polyclonal rabbit antibody for mouse extracellular E-Cadherin domain were from R&D Systems Europe. Monoclonal mouse antibodies for human extracellular and polyclonal rabbit antibody against human cytoplasmic E-Cadherin domain were from Cell Signalling Technology (Ozyme) and Takara (Cambrex Bio Science). Monoclonal mouse antibody against human glyceraldehyde 3-phosphate dehydrogenase (GAPDH) was from Chemicon International (Abcys). Secondary FITC-labelled or HRP conjugated polyclonal goat antibody against mouse immunoglobulin and secondary FITC-labelled or HRP conjugated polyclonal swine antibody against rabbit immunoglobulin were from Dakocytomation (Trappe’s). All tissue culture reagents and other chemicals were reagent grade and purchased from Invitrogen or Sigma-Aldrich.

### Human samples, mice, cells, and bacteria

Bronchoalveolar lavage (BAL) specimens. Remainders of BAL fluids were obtained in conjunction with a prior study of patients with COPD exacerbation [29, 30].

Mice. NE-deficient mice (NE−/−) were generated by targeted mutagenesis [8]. NE−/− and wild type (WT) mice (C57Bl/6 J, 8–10 weeks old) were housed in a pathogen-free facility with 12 h light/dark cycle and provided with food and water ad libitum.

Cells. Human bronchial epithelial cell line 16HBE (kindly provided by Dr. Gruenert, University of California at San Francisco, San Francisco, CA) and mouse alveolar cell line MLE15 (kindly provided by J. Wittset, University of Cincinati, Cincinati, OH) were cultured to confluence in DMEM (for 16HBE) or RPMI 1640 (for MLE15) culture media as previously described [31, 32]. Prior to any treatment, cells were washed three times with sterile phosphate-buffered saline (PBS) and cultured for 1 h in the absence of FCS.

Polymorphonuclear neutrophils (PMNs) were isolated from mouse WT femur and tibia tips-derived bone marrow as previously described [33]. A Percoll gradient was employed to separate morphologically mature neutrophils, which were used immediately. Purified neutrophils represented >95 % of the cell population and >98 % were viable as judged by differential counting and trypan blue dye exclusion respectively.

Bacteria. In this work, we used *Pseudomonas aeruginosa* H103 (kindly provided by R. Hancock, University of British Columbia, Vancouver, Canada). Bacteria were grown aerobically to late exponential phase (3 h), washed twice, and resuspended in 1 ml of PBS (pH 7.4). The optical density (OD) of bacterial culture was determined at 600 nm (OD) (OD 1 ≈ 10^9^ bacteria/ml).

### Exposure of epithelial cell protein extracts to NE

Media of cell cultures were removed and confluent cells were scraped. Proteins were extracted from cell pellet using RIPA buffer and quantified as previously described [34]. Next, equal protein aliquots (10 μg) were incubated alone or in the presence of varying concentrations of NE, CG or PR3 at 37 °C for designated periods of time. The reactions were carried out in a 20 μl volume in PBS at pH 7.4, which should approximate the pH in the extracellular milieu of the lung and corresponds to pH optimum of NE. In parallel experiments, NE (50 nM, highest concentration) was preincubated with SLPI (100 nM) at 37 °C for 5 min prior to addition to cell protein extracts.

### Exposure of epithelial cells to NE

Confluent 16HBE epithelial cell monolayers were cultured alone or in the presence of designated concentration of purified NE for a defined period of time [35]. In parallel, NE was preincubated with SLPI at 37 °C for 5 min prior to addition to cells. At the end of treatment time, culture supernatants were collected, centrifuged to remove cell debris, and acetone-concentrated. Cells were scraped and proteins were extracted and quantified as described above. Equal aliquots of culture supernatants or lysate proteins (10 μg) were resuspended in PBS, and subjected to SDS-PAGE and Western blotting as described below.

### Immunofluorescence microscopy

MLE-15 epithelial cells were grown on cover slips to confluence. Next, cell monolayers were cultured alone or in the presence of designated concentration of purified NE for a defined period of time. In parallel experiments, mouse PMNs were added to MLE-15 epithelial cells at a ratio 1:10 (epithelial cell:PMN). Prior to addition to epithelial cells, neutrophils were first primed and stimulated by addition of LPS (10 μg/ml) and formyl-methionyl-leucyl-phenylalanine (fMLP, 1 μM) [34]. NE release from activated cells was examined using NE specific chromogenic peptide substrate. Under these experimental conditions, over 80 % of neutrophils were still alive upon addition to epithelial cells, as judged by trypan blue dye exclusion. However, their viability was compromised 6 h after co-culture since we could barely detect their nuclei by staining with TO-PRO-3 iodide.

Six hours post-treatment, cover slips were processed for immunofluorescence microscopy. Briefly, cells were fixed for 10 min in 3 % (w/v) paraformaldehyde. Nonspecific binding was blocked with 3 % bovine serum albumin in PBS for 30 min, and cells were incubated with polyclonal antibody specific for mouse extracellular E-cadherin domain (dilution 1:750) for 1 h at room temperature. After washing, samples were FITC-labelled secondary antibody-immunostained (dilution 1:2000) for fluorescence microscopy [34]. The nuclei were stained with TO-PRO-3 iodide (Molecular Probes) for 10 min. Following a final gentle wash, cells were mounted in Vectashield (Vector Laboratories) on slides using Secure Seal imaging spacers (Sigma). Samples were examined using a LSM 510 Meta laser scanner microscope (Carl Zeiss Inc., Thornwood, New York, USA). Equivalent concentrations of preimmune serum were used as a negative control. Fluorescence intensity was estimated by determination of the average area of stained regions [35, 36]. Briefly, 15 randomly digitized images representative of each condition of the experiments were captured in a blinded manner (A.B.) and analysed using the image analysis software ImageJ (NIH, Bethesda, MD).

### Time-lapse videomicroscopy

First, confluent monolayers of 16HBE epithelial cells were first subjected to immunofluorescence staining as described above. Cells were incubated with human anti-E-Cad specific antibody against the extracellular domain (dilution 1:750) followed by FITC-labelled secondary antibody (dilution 1:2000). Next, cell monolayers were incubated alone or in the presence of NE (200 nM). In parallel experiments, cells were exposed to NE that was pretreated with SLPI (400 nM) or SLPI alone.

Cell culture plates were transferred immediately to Zeiss IM35 inverted microscope (Zeiss, Oberkochen, Germany) equipped with an incubation chamber maintained at 37 °C with 5 % CO_2_ in a wet atmosphere. Video recording was performed using a Panasonic WVCD51 digital camera (Osaka, Japan) controlled by a Sparc 2 Sun workstation (Sun Microsystems, Mountain View, CA) with a video board (Parallax Graphics, Santa Clara, CA) [31]. Images of untreated or NE-treated cells were captured every 15 min for 6 h using 10X magnification to analyse at least 100 cells per field of view.

### Mouse model of pneumonia

Mice were intranasally challenged with *P. aeruginosa* bacteria or sterile saline. Briefly, mice were anesthetized followed by intranasal administration of 50 μl of sterile PBS or containing a sublethal dose of bacteria (4×10^6^ colony forming units (CFUs)/per mouse, which we have previously shown to injure acutely mouse lungs [34].

Groups of mice (*n* = 4/genotype/time point) were sacrificed at designated time points, and their lungs were lavaged in situ using Hank’s balanced salt solution (pH 7.4), cycled in three times. Identical recoveries of lavages (700 ml/mouse) were obtained for each of the experimental groups. Cell counts from BAL fluids were immediately performed by hemocytometer and aliquots of BAL fluids were cytospun and Wright-stained for differential counting (Thermo Shandon and Fisher Scientific). Next, the remaining BAL samples were centrifuged for 10 min at 4 °C to remove cells, aliquoted and snap-frozen until use. Mouse lungs were processed for histologic analyses as previously described [35].

### Western blotting

Equal proteins aliquots derived from cell cultures and murine or human BAL fluids were resolved on 12 % SDS-polyacrylamide gels and transferred to polyvinylidene difluoride membranes (Millipore Corp., Bedford, MA). Of note, unlike murine BAL fluids, human BAL (5 ml) were concentrated by lyophilisation prior to determination of protein concentration [35]. Blots were incubated with the indicated antisera specific to extracellular or cytoplasmic E-cad domains or NE (dilution for E-cad antibodies 1:1000 and dilution for NE 1:2500) [35]. Membranes were subsequently incubated with an appropriate dilution of HRP-linked secondary IgG (dilution 1:2000) in blocking buffer. Immunoreactive fragments were visualized by enhanced chemiluminescence (ECL, Amersham Biosciences). Labelled proteins or fragments were detected with the Molecular Imager ChemiDoc XRS System and quantified using Quantity One 1-D Analysis Software (Biorad, Marnes-la-Coquette, France).

### Neutrophil elastase activity

Neutrophil elastase activity in cell-free BAL fluids was assessed using conventional chromogenic peptide assays [35]. Briefly, BAL aliquots (100 μl) were incubated with NE peptide substrate Meo-Suc-Al-Al-Pro-Val-pNA (0.2 mM) for 60 min at 37 °C in a total volume of 200 μl Tris-NaCl buffer (0.1 M Tris and 1 M NaCl, pH 7.4). The reactions were then spun (30 s, 12,000 rpm), and changes in the absorbance of the supernatants were determined spectrophotometrically at λ 410 nm.

### Immunohistochemistry

Immunostaining on lung tissue sections was performed as previously described [34]. Sections were incubated with antibodies specific for mouse E-cad (dilution, 1:750) at 4 °C overnight. Next, samples were incubated for 20 min with biotinylated secondary antibody and labelled with HRP-conjugated streptavidin. Immune complexes were visualized using 3’3-diaminobenzidine (DAB) (Biocare Medical) as substrates for HRP and counterstained with Mayer’s hematoxylin. Slides were examined using a LSM 510 Meta laser scanner microscope.

### Lactate dehydrogenase (LDH) activity assay

Changes in LDH activity were used as a marker for cytotoxicity and were assessed in cell-free BAL fluids by the LDH kit following the manufacturer’s recommendations (Sigma-Aldrich) [37]. LDH catalyses the oxidation of lactate to pyruvate. This reaction is coupled with reduction of NAD to NADH, which is followed spectrophotometrically at 340 nm. The LDH activity is proportional to the rate of absorbance changes. Briefly, 100 μl of BAL samples were added to 900 μl LD-L reagent and changes in absorbance were recorded over 3 min.

### Albumin concentration

The albumin level changes were examined using the bromocresol green assay (BCG, Sigma-Aldrich), according to the manufacturer’s recommendations [37]. Briefly, 100 μl of samples or standards were incubated with 900 μl of BCG substrate for 60 s at room temperature. Changes in absorbance were recorded by spectrophotometer at 628 nm and were proportional to the albumin concentrations in the samples.

### Statistics

Densitometric quantification of western blots was performed with the AIDA software (Raytest, Staubenhardt, Germany). Data were analysed by Kruskall-Wallis and Mann–Whitney tests with the StatView software (SAS Institute Inc., Cary, NC, USA).

## Results

### NE degrades E-cad in an enzyme dose- and incubation time-dependent fashion

**Fig. 1:**
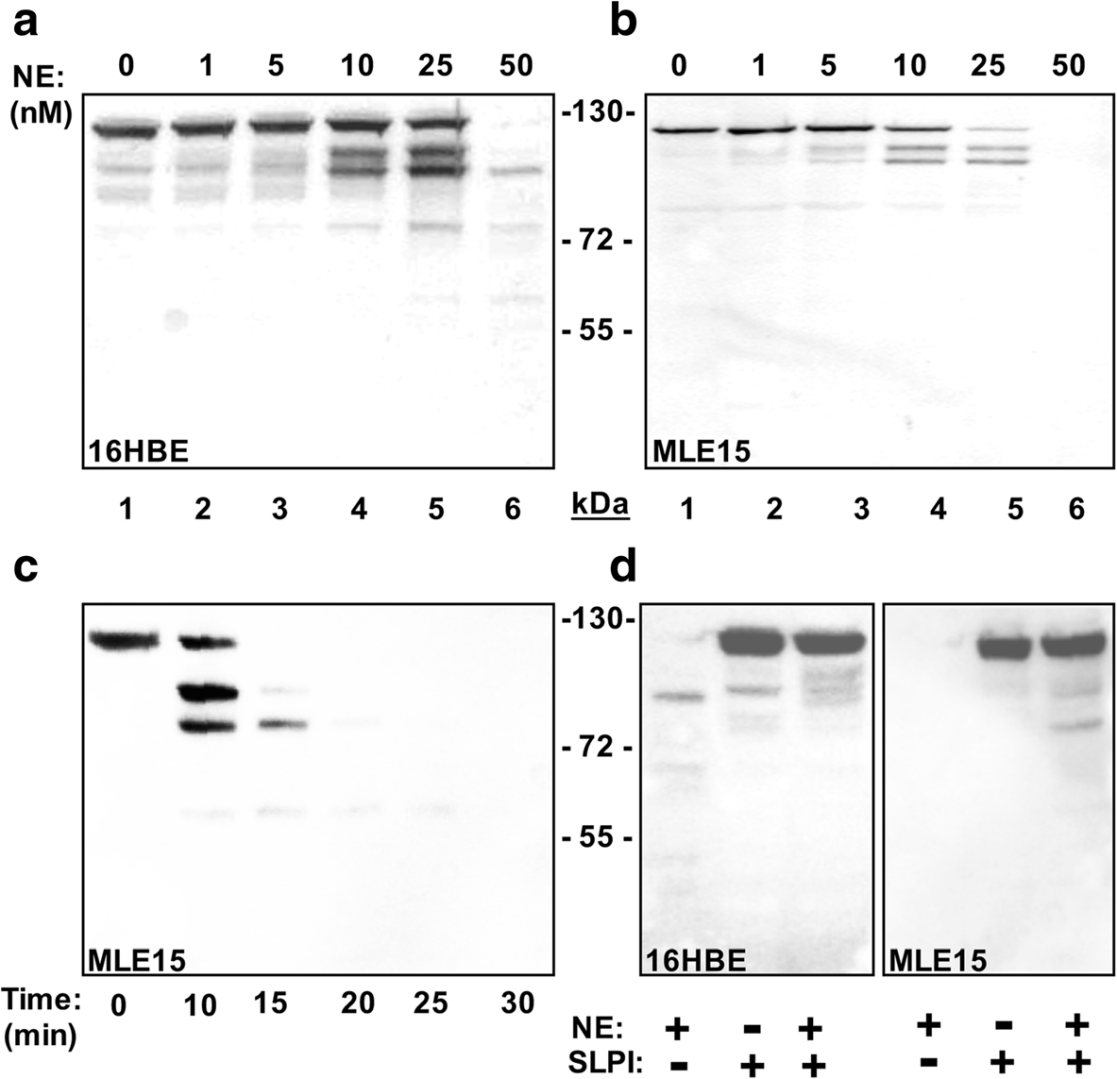
Degradation of E-cad is NE concentration and incubation time dependent. Epithelial cell protein extracts containing E-cadherin (10 μg) were incubated alone or in the presence of varying concentrations of NE for various time periods. The reactions were resolved by SDS-PAGE under reducing conditions and visualized by immunoblotting. **a-b**, incubation with NE (0, 1, 5, 10, 25, 50 nM) for defined time point (30 min) resulted in an enzyme dose-dependent degradation of E-cad derived from 16HBE (**a**) or MLE15 (**b**) epithelial cell extracts. **c**, Incubation of MLE15 epithelial cell extracts with NE (50 nM) for defined time points (0, 10, 15, 20, 25, 30 min) resulted in stepwise degradation of E-cad. **d**, E-cad cleavage was largely abrogated in both 16HBE and MLE15 protein extracts when NE (50 nM) was pre-incubated with the physiologic serine proteinase inhibitor SLPI (100 nM). Molecular weight (MW) (kDa) standards are between panels. The findings are illustrative of at least three independent experiments

To determine whether E-cad potentially represents a substrate for NE, we incubated cell protein extracts containing E-cad with purified NE. We used protein extracts derived from bronchial cell line 16HBE or alveolar epithelial-like cell line MLE15. Various NE concentrations can be encountered in vivo including very high concentrations near sites of granule exocytosis [38]. Accordingly, we examined a wide range of NE concentrations (1, 5, 10, 25, and 50 nM) against a fixed cell protein extract for varying incubation times. As judged by Western blotting using specific polyclonal antibodies against human or mouse extracellular E-cad domain, identical fragmentation was observed with both human and mouse cell line protein extracts in a manner dependent of NE dose and incubation time (Fig. 1a-c). Progressive cleavage of E-cad started with enzyme concentration as low as 5 nM. Also, NE catalysed rapid and complete cleavage of intact E-cad (100 % of E-cad was completely degraded by 50 nM of NE within 15 min) (Fig. 1c). Of interest, identical degradation patterns were obtained when incubations were performed in PBS (pH 7.4) or Tris-buffered saline (data not shown). Significantly, E-cad degradation was prevented when NE was preincubated with the specific serine protease inhibitor SLPI indicating that the catalytic activity of the enzyme is required to cleave E-cad (Fig. 1d). Of interest, cleavage of E-cad was also observed in the presence of the other two neutrophil serine-proteases CG and PR3, but NE was by far the most potent degrading protease (Additional file 1). In the subsequent experiments, we focused on NE for further characterization of E-cad cleavage.

### NE degrades cell-associated E-Cad and generates a distinct extracellular fragment

**Fig. 2:**
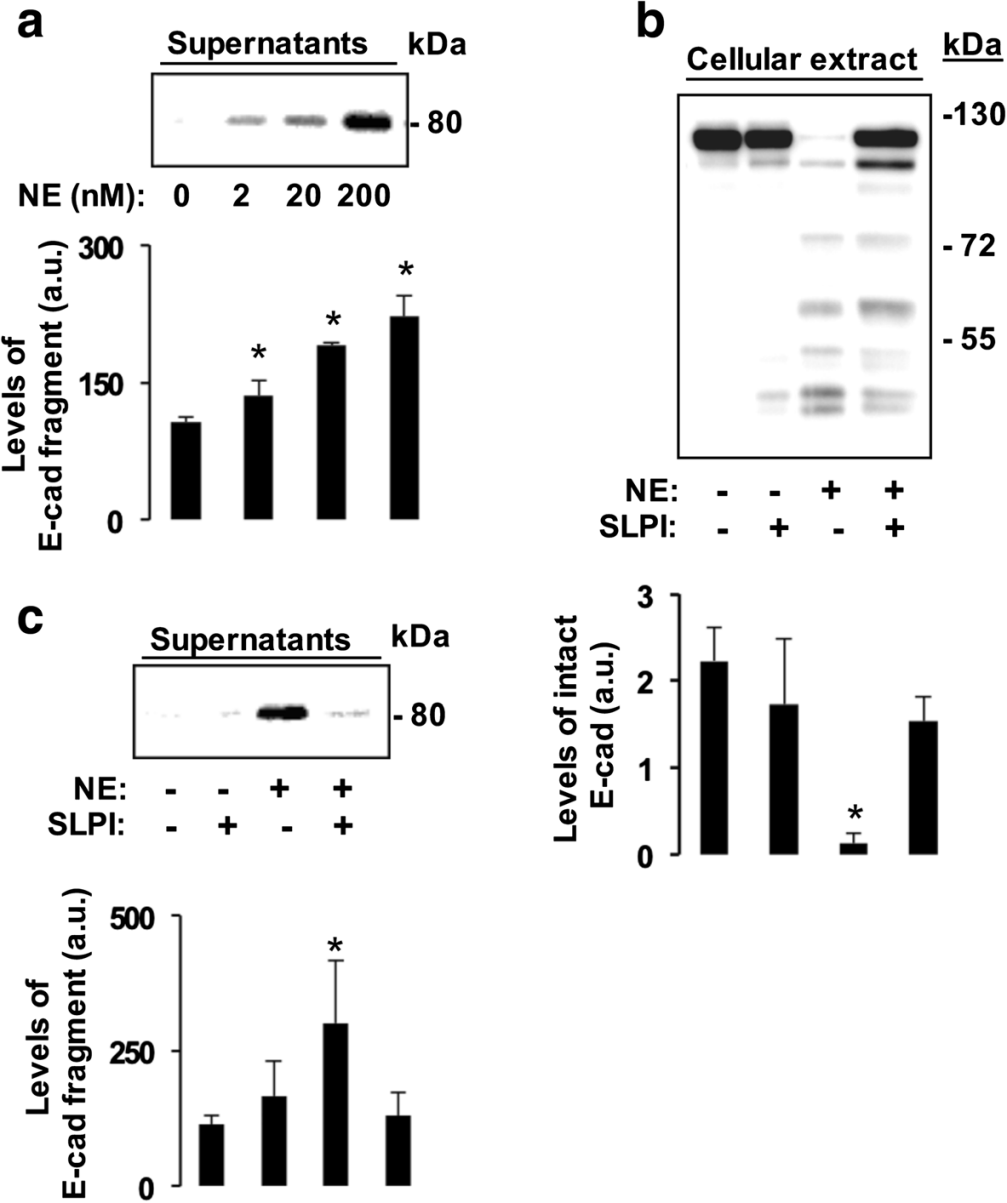
NE degrades cell-associated E-Cad and generates a distinct extracellular fragment. Confluent 16HBE cells were left untreated or treated with varying concentrations of purified NE (0, 2, 20, or 200 nM) for 6 h. Next, equal protein aliquots from culture supernatants and cell lysates (10 μg) were subjected to SDS-PAGE and immunoblotting using antibodies raised against N- and C-terminal parts of E-cad respectively. **a**, left panel. Anti-E-cad N-terminal antibody revealed a progressive increase of a distinct N-terminal fragment of about 80 kDa that paralleled the increase of NE concentration. **a**, lower panel, densitometric analysis confirms increased levels of E-cad fragment. Data are mean values ± SD. *p < 0.05; Kruskall-Wallis test. **b**, right panel. Loss of intact E-cad was achieved with NE at 200 nM. Of note, anti-E-cad C-terminal antibody detected varying fragments. NE inhibition with SLPI (400 nM) prevented degradation of cell-associated E-cad. **b**, lower panel, densitometric analysis found low levels of intact E-cad when cells were exposed to NE alone. However, preincubation of NE with SLPI prevented considerably such degradation. Data are mean values ± SD. *p < 0.05; Mann–Whitney test. **c**, left panel. Immunoblotting analysis of condition media from B with an anti-E-cad N-terminal part confirmed the generation of the distinct 80 kDa degradation product, which paralleled the loss of intact E-cad. **c**, lower panel, densitometric analysis found increased level of E-cad fragment concomitant with low levels of intact E-cad when cells were exposed to NE alone in B. However, preincubation of NE with SLPI prevented considerably E-cad degradation. MW standards are on wright. a.u., arbitrary unit. Data are mean values ± SD. * corresponds to *p* < 0.05 for cleaved versus intact E-cad; Mann–Whitney test. Experiments were repeated three times with similar findings

The ability of NE to degrade E-cad was next examined in cell culture system. After exposure of cells to increasing concentrations of NE for a fixed time period, both cell lysates and condition media were analysed by Western blotting. As shown in Fig. 2a-left panel, using an antibody raised against human N-terminal part of E-cad, levels of a distinct cleavage product of about 80 kDa increased progressively in culture supernatants in function of NE concentration coinciding with the disappearance of E-cad protein in cell lysates (Additional file 2). Using an antibody raised against the C-terminal part of E-cad, complete degradation of the protein in cell lysates was achieved at 200 nM, four fold higher than that used with protein extracts of Fig. 1 (Fig. 2b, left panel). Analysis of condition media with an antibody against the N-terminal part confirmed loss of intact E-cad, which paralleled the appearance of the distinct 80 kDa degradation fragment (Fig. 2c, left panel). As in Fig. 1, inhibition of NE by SLPI, prevented cell-associated E-cad degradation and SLPI alone had no effect (Fig. 2b and c). All these data were further supported by densitometric analyses (Fig. 2a - c; right panels).

### Alteration of epithelial cell monolayer following exposure to purified NE or activated neutrophil-derived NE

**Fig. 3:**
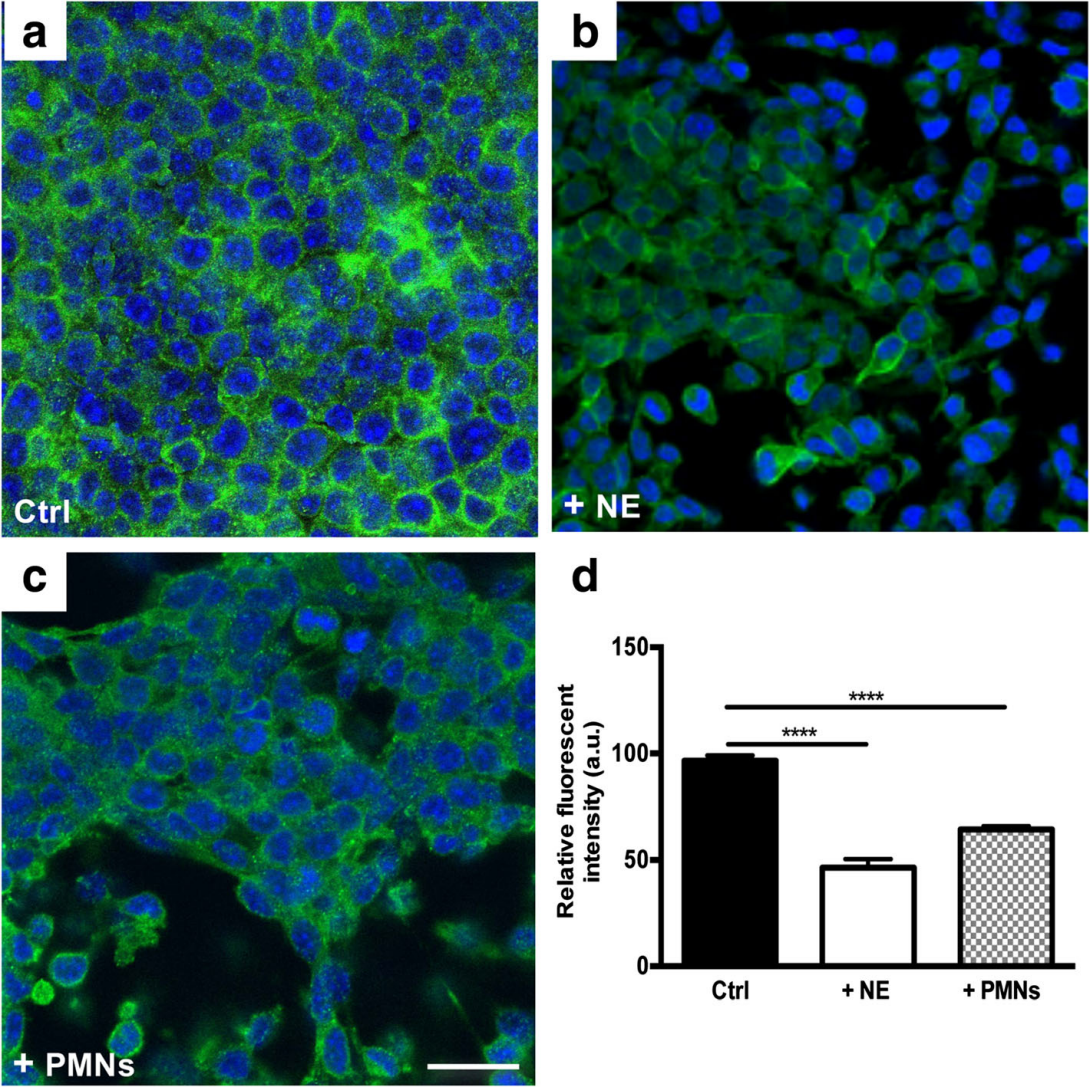
Purified NE or activated neutrophils alters epithelial monolayer integrity. Confluent cultured MLE15 cells were exposed to purified or activated neutrophils for 6 h. **a**, immunofluorescence staining of untreated cell monolayer with N-terminal E-cad antibody found strong immunoreactive E-cad surrounding cells. **b**, exposure of cells to purified NE (200 nM) resulted in considerable loss of E-cadherin immunostaining and disruption of cell-cell adhesion. **c**, activated neutrophils alter epithelial cell layer integrity with the appearance of patchy gaps. The number of neutrophils added to cells was calculated to yield concentration of liberated NE closely equivalent to that of purified NE. **d**, fluorescence quantification of cultured cells. Note NE-treated cells or cells co-cultured with neutrophils showed significantly less staining than untreated cells. Data are presented as means ± SEM per experimental condition. **** correspond to *p* < 0.0001 for conditions of cell treatment with NE or PMNs versus control. a.u., arbitrary unit. Scale bar, 120 μm. Experiments were repeated three times with similar observations

Next, to examine the impact of NE-mediated degradation of E-cad on the integrity of epithelial cell monolayer, purified or activated neutrophil-derived NE (free and/or cell-bound) were added to cultured cells. Immunofluorescence staining of untreated cell monolayer with N-terminal E-cad antibody found strong immunoreactive E-cad surrounding cells, consistent with its known membrane localization that confers cells with tight adhesion (Fig. 3a). Incubation with purified NE (200 nM), however, induced loss of E-cadherin immunostaining resulting in the appearance of patchy gaps within the monolayer (Fig. 3b).

We then sought to determine whether secreted NE by activated neutrophils mediates similar effects. The number of neutrophils added per well was calculated to yield concentrations of liberated NE approximately comparable to that shown in Fig. 3b [34]. As for NE, addition of activated neutrophils resulted in loss of epithelial cell layer integrity suggesting the most likely implication of this protease (Fig. 3c). Immunoblotting experiments on protein extracts of cells or culture supernatants found loss of intact E-cad and generation of cleaved product (data not shown); findings consistent with those in Fig. 2.

### Spatio-temporal dynamics of NE-mediated disruption of epithelial cell monolayer

**Fig. 4:**
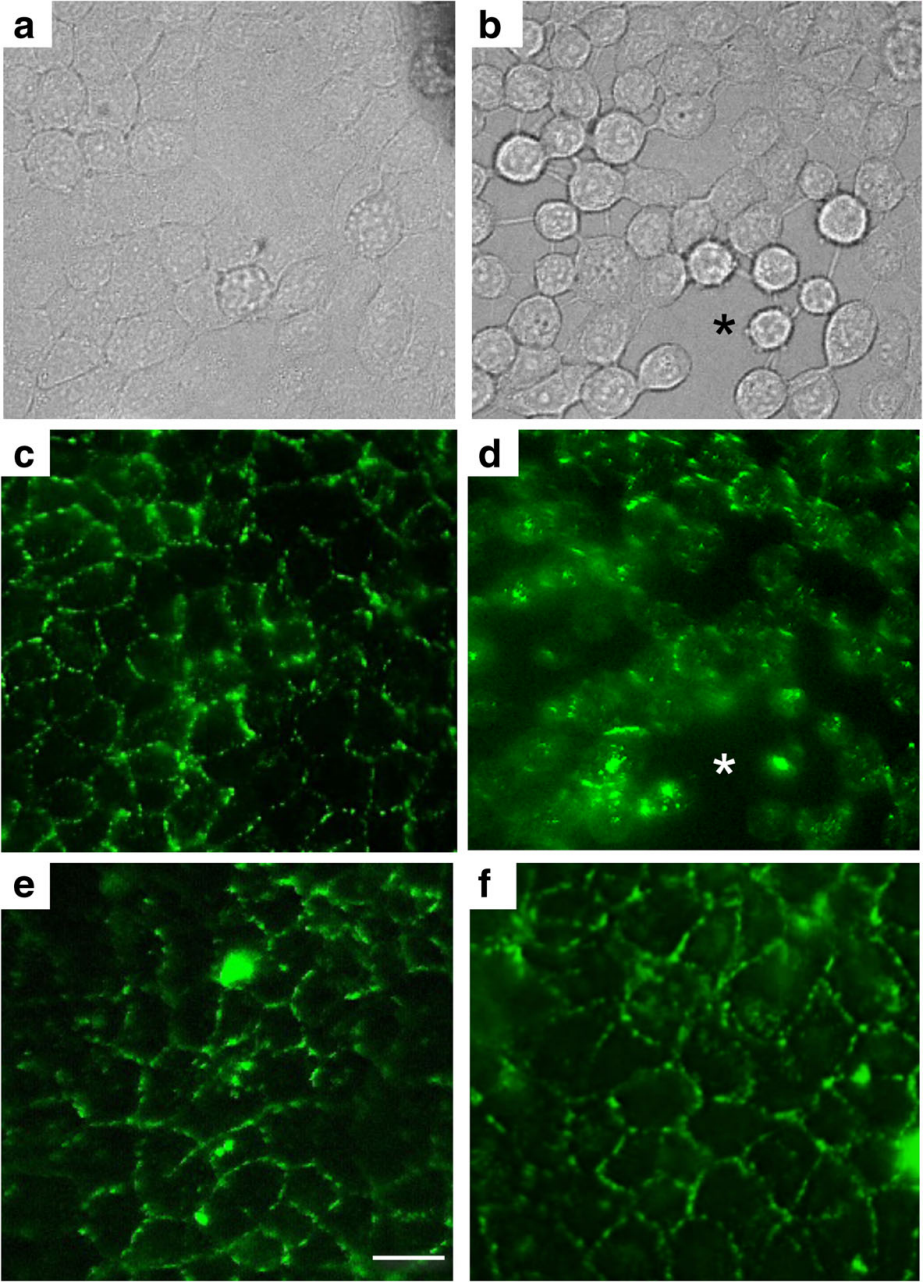
Spatio-temporal changes of epithelial cell monolayer following treatment with NE. Confluent monolayers of 16HBE epithelial cells were incubated alone or in the presence of defined NE concentration (200 nM). Briefly, cells were first subjected to immunofluorescence staining of E-cad as described in material and methods. Next, cell monolayers were left untreated or exposed to NE (200 nM) alone or pre-incubated with SLPI (400 nM). Video recording was performed for 6 h. **a-b**, Representative phase contrast images of untreated (**a**) or NE-exposed (**b**) 16HBE cells. Unlike control cells with flat polygonal morphology, NE-treated cells detached from each other and displayed rounded morphology. **c-d**, Representative immunofluorescence images of untreated or NE-exposed 16HBE cells. Immunofluorescent staining showed E-cad surrounding uniformly cohesive cells (**c**). In contrast, cells exposed to NE lost their immunostaining for E-cad coinciding with the appearance of intercellular gaps (**d**). Of note, NE preincubated with SLPI (**e**) or SLPI alone (**f**) had less striking effect on E-cad distribution and monolayer integrity. Asterisks depict formed gaps. Scale bar = 40 μm. Experiments were repeated twice with similar observations


To follow changes in epithelial cell monolayer integrity in association with NE-mediated degradation of E-cad, we used time-lapse videomicroscopy approach. Cell behaviour was analysed first by phase-contrast image recordings. Initially, cells displayed a flat polygonal morphology (Fig. 4a). Following addition of NE, the morphology of cells changed and became rounded due to detachment of cells from each other, in distinct areas (Fig. 4b and an additional movie file shows this in more detail [see Additional file 3: Movie S1]). At the end of recording time, considerable number of cells was floating in culture medium. Of note, there were areas of the cell monolayer that were not affected by morphologic changes.

Additional file 3: Videomicroscopy 1 (Bright field, addition of NE to cultured cells). (MOV 2864 kb)


Using immunofluorescence staining combined with time-lapse videomicroscopy, the pattern of E-cad in untreated cells was similar to that seen in Fig. 3a throughout the recording time. Indeed, E-cad was uniformly distributed around cohesive cells (Fig. 4c). In contrast, cells exposed to NE exhibited a dramatic decrease of fluorescence intensity over recording time, concomitant with the appearance of rounded cells and marked intercellular space enlargement (Fig. 4d and an additional movie file shows this in more detail [see Additional file 4: Movie S2]). There were less striking morphologic changes and variations in fluorescence intensity in plate wells treated with either NE that was preincubated with SLPI or SLPI alone (Fig. 4e and f and additional movie files show this in more detail [see Additional file 5: Movie S3 and Additional file 6: Movie S4]).


Additional file 4: Videomicroscopy 2, (Immunofluorescence, addition of NE to cultured cells). (MOV 1338 kb)



Additional file 5: Videomicroscopy 3, (Immunofluorescence, preincubation of NE with SLPI prior to addition to cultured cells). (MOV 2194 kb)



Additional file 6: Videomicroscopy 4, (Immunofluorescence, addition of SLPI alone to cultured cells). (MOV 2084 kb)


### In vivo detection of E-cad and NE

**Fig. 5:**
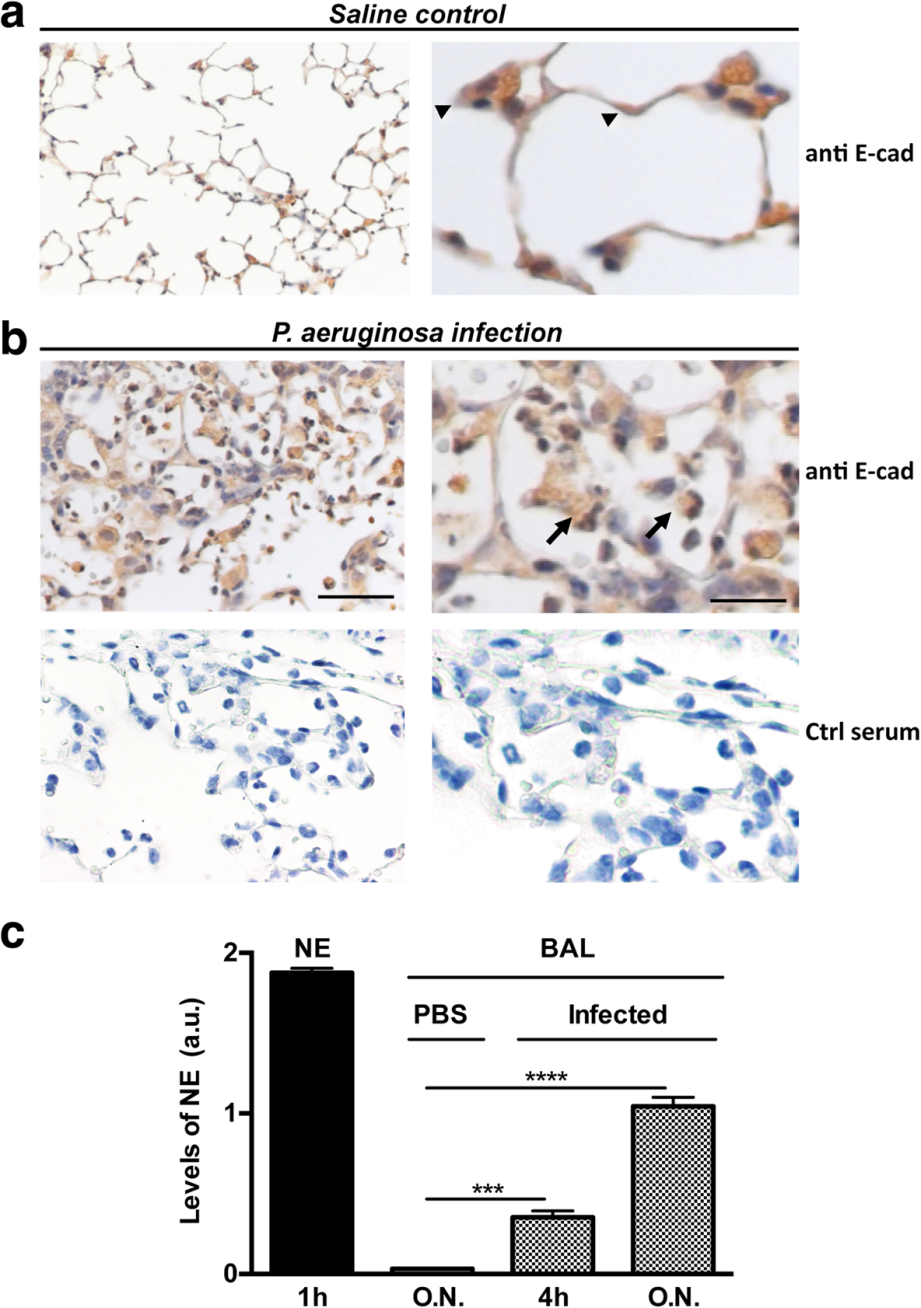
In vivo detection of E-cad and NE. **a**, Lung tissue sections from saline control mice were immunostained for mouse E-cad. Under physiologic conditions, E-cad is restricted to epithelial lining (arrowheads), consistent with the known localization of E-cad expression. **b**, Representative mouse lung tissue section following O.N. challenge with *P. aeruginosa*. **b**, upper panels, Immunostaining for E-cad detected the antigen within inflamed airspaces and in close spatial contact with the cellular infiltrates (arrows). **b**, lower panels, absence of E-cad staining in the presence of preimmune serum. **c**, absence of staining was seen when the primary antibody was replaced with the preimmune serum. **c**, Enzymatic activity assay detected free active NE in cell-free BAL. Consistent with the neutrophilic response to *P. aeruginosa*, increasing levels of active NE were detected in cell-free BAL fluids following O.N. infection (infected tissues). No NE activity was detected in mice challenged with sterile saline (PBS). Purified active NE (100 ng) was used as control. Data were consistent in all challenged mice (bars ± SEM; *** and **** correspond to *p* < 0.001 and *p* < 0.000 respectively for infection versus PBS). O.N. corresponds to 24 h. Scale bar of left images, 150 μm. Scale bar of right images, 45 μm

Next, we determined whether NE mediates E-cad degradation in injured lungs using our previously described mouse *P. aeruginosa* pneumonia model [34]. To circumvent the confounding effect of *P. aeruginosa* metalloelatase, known to degrade a variety of host molecules, we employed *P. aeruginosa* strain H103, which lacks this enzyme [39]. Immunostaining of lung sections from unchallenged control wild type mice found E-cad expression restricted to epithelia, consistent with the known sites of E-cad localization (Fig. 5a) [25]. However, O.N. post-infection with *P. aeruginosa*, strong E-cad immunostaining was observed in both epithelial lining and intraalveolar exudates (Fig. 5b, upper panels). No staining was seen when the primary antibody was replaced with the preimmune serum (Fig. 5b, lower panels). Next, we analysed lung tissues of control and infected animals to look for evidence of simultaneous presence of active NE and E-cad. NE activity assay in BAL fluids detected significant amounts of NE suggesting the presence of active neutrophil-derived NE within the alveolar spaces (Fig. 5c). Concomitant with E-cad degradation and active NE, analysis of the inflammatory response in BAL fluids found a massive cellular recruitment predominated with neutrophils and increased albumin level and LDH release (Additional file 7, [34, 40]).

### NE contributes to degradation of endogenous E-cad in the setting of lung injury

**Fig. 6:**
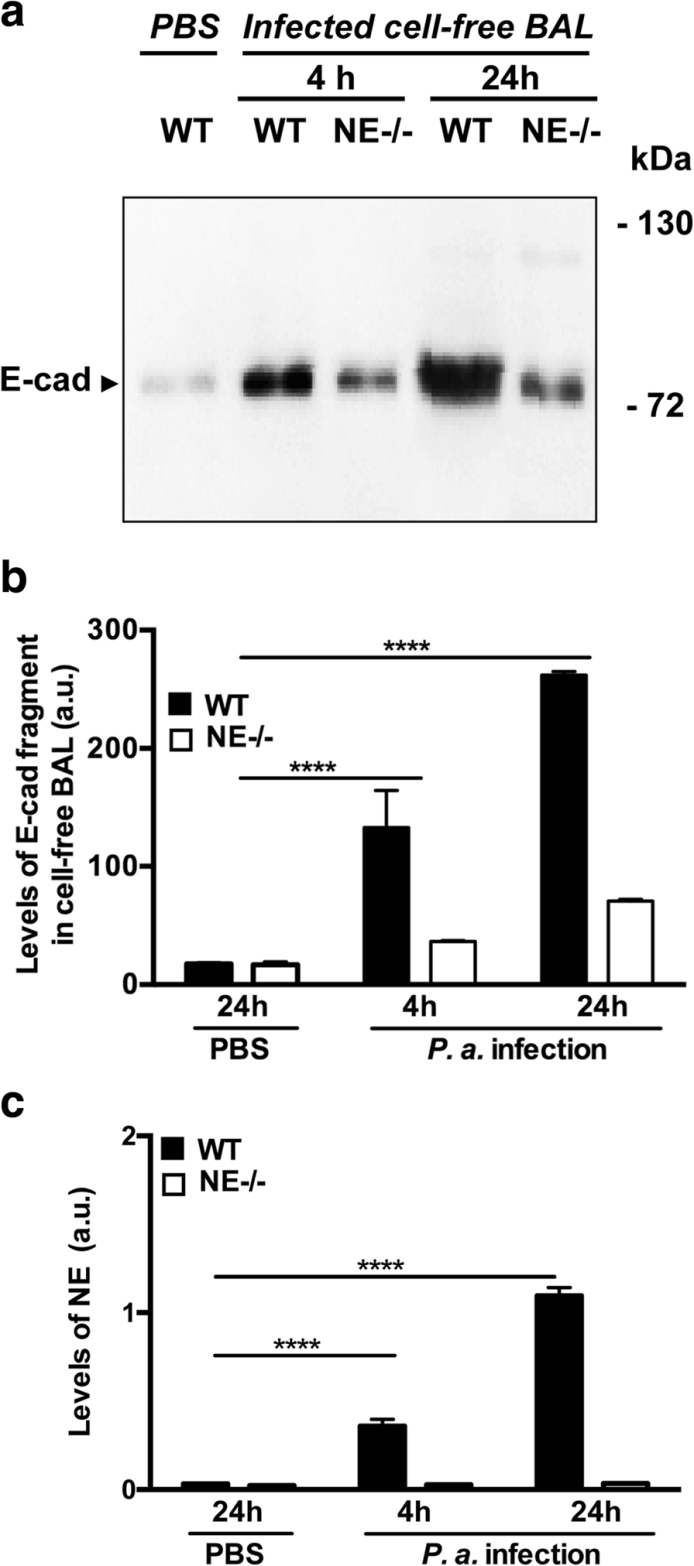
In vivo contribution of NE to degradation of endogenous E-cad. **a**, Representative image of equal BAL fluid protein aliquots (in 20 μl) from control mice and mice i.n. challenged with P. aeruginosa for 4 h or O.N. that were reduced and processed for immunoblotting using E-cad N-terminal antibody. Both 4 and O.N. time points revealed a gradual degradation of endogenous E-cad with generation of a distinct ~80 kDa cleavage fragment that migrated similar to the cleavage fragment generated by purified NE. Note cell-free NE−/− BAL fluids showed less cleaved E-cad fragment by comparison to cell-free WT BALs. **b**, Densitometric analysis of immunoblot images corresponding to all cell-free BAL fluids per group, genotype and condition confirmed the protein profile of Fig. **a. c**, enzymatic activity assay analysis revealed the presence of active NE in cell-free WT, but not NE−/−, BAL fluids that became abundant over time (O.N.). Data were similar in all challenged mice and mouse experiment was repeated once with reproducible findings (bars ± SEM; **** corresponds to *p* < 0.0001for infection versus PBS)

To assess the specific role of NE in neutrophil-mediated degradation of E-cad, WT and NE-deficient (NE−/−) mice were i.n. challenged with *P. aeruginosa* as described above and sacrificed 4 and 24 h post-challenge. As shown in Fig. 6a and b, immunoblotting of infected cell-free WT and NE−/− BAL fluids and densitometric analysis revealed gradual generation of endogenous cleavage product in function of time, with similar ~80 kDa size to that generated by purified NE. More importantly, NE−/− cell-free BAL fluids showed less degradation of E-cad as compared to WT cell-free BALs. NE activity assay analysis revealed the presence of active NE in WT, but not NE−/−, cell-free BAL fluids that became abundant at time point 24 h (Fig. 6c). Thus, our findings demonstrate that NE contributes to efficient cleavage of E-cad. Of note, low levels of E-cadherin extracellular domain were also seen in PBS-challenged mice. This suggests that either a basal level of E-cadherin is constitutively shed from the epithelium or as a consequence of the shear stress of the in and out cycling of PBS during lung lavage.

### Detection of both cleaved E-cad and NE in BAL of patients with exacerbated COPD

**Fig. 7:**
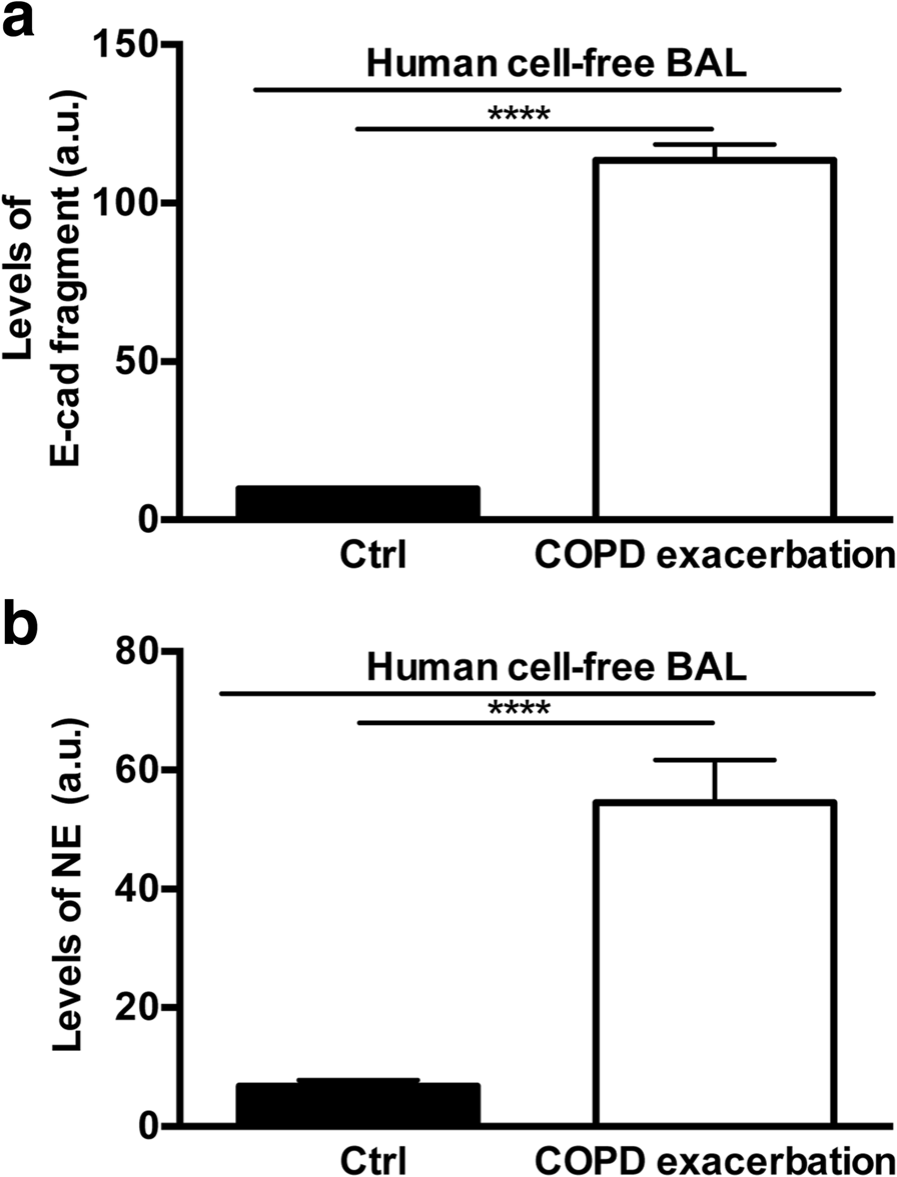
Detection of cleaved endogenous E-cad coincides with NE in human diseased lungs. Equal protein aliquots (in 20 μl) of concentrated cell-free BAL derived from patient with COPD exacerbation (*n* = 5) were subjected to SDS-PAGE and immunoblotting using antibodies raised against human E-cad extracellular domain and NE respectively. **a**, densitometric analysis found increased level of immunoreactive E-cad fragment (~80 kDa). **b**, densitometric analysis revealed the presence of immunoreactive NE in the same samples of (**a**). Experiments were repeated three times with similar results (bars ± SEM; **** corresponds to *p* < 0.0001 for COPD exacerbation versus control)

To determine the relevance of cell and animal data to human lung diseases, we looked for evidence for the presence of both degraded E-cad and NE in cell-free BAL fluids obtained from exacerbated COPD patients characterized by an abnormal influx of neutrophils. As shown in Fig. 7a, there was a marked increase in the levels of ~80 kDa immunoreactive E-cad fragment in all cell-free BAL samples, a finding that corroborated our cell culture and mouse data. Next, after stripping and immunoblotting of the same Western blot membranes for NE, densitometric analyses revealed the presence of increased NE levels as well confirming the predominance of active neutrophils in the samples (Fig. 7b). Detection of both cleaved E-cad and NE suggests, thus, that both proteins are present in inflamed human lungs and support the hypothesis that NE could target E-cad in vivo contributing potentially to the setting of lung inflammation and injury.

## Discussion

That E-cad is susceptible to NE has been previously reported in limited cell culture [27, 41, 42] and experimental model studies [24–26]. The originality of our work is several folds. As expected, the inflammatory response of our mouse model of acute *Pseudomonas* pneumonia was characterized by increased alveolocapillary permeability, neutrophil influx, and LDH. Using genetically engineered mice deficient in NE in this experimental model, we provide compelling evidence that NE contributes substantially to E-cad degradation in injured lungs. Videomicroscopy approach showed that the spatial and temporal NE-mediated degradation of E-cad was accompanied with the abrogation of this latter’s cell-cell adhesion function. More importantly, the presence of both degraded E-cad and NE was detected in COPD exacerbation, a clinical manifestation characterized by predominant neutrophilic inflammatory response. It must be noted that a limited number of human samples (*n* = 5) have been examined in this study and to further document the generality of our findings investigation of a large number of human BAL fluids is warranted. Altogether, these data suggest the likelihood contribution of NE-mediated degradation of E-cad in the development of inflammation and tissue destruction in the setting of neutrophil-rich lung diseases. Of importance, the concentrations of NE used in both in vitro and cell culture studies are relevant since they can be even exceeded in pulmonary diseases [43, 44]. Also, in the mouse experimental model, E-cad degradation was not completely prevented in the absence of NE suggesting contribution of other proteases. In this regard, activated PMNs release two other members of neutrophil serine protease family, cathepsin G (CG) and proteinase 3 (PR3) known to share the same conserved catalytic cleft as NE [5]. Interestingly, our in vitro data showed that while the two other members of neutrophil serine protease family, cathepsin G (CG) and proteinase 3 (PR3) degraded E-cad, NE was the most potent degrading enzyme. The relative importance of CG and PR3 in cleaving E-cad would be, however, best defined using mice deficient in these proteases.

In general, epithelial cell-cell adhesion proteins are grouped in three categories (from apical to basal side): tight junctions (e.g., ZO-1 and occludin), adherens junctions (E-cad) and desmosomes. There is a large body of evidence indicating that E-cad represents a key protein for the establishment and maintenance of cohesive epithelium. Indeed, the protein mechanically ensures tight adhesion of cells [45]. It is required for the formation of other junctional complexes such as tight junctions [46]. E-cad regulates cell proliferation and differentiation [47]. Furthermore, E-cad plays a modulatory role in host immune responses by regulating the expression of growth factors and proinflammatory mediators [48]. Consequently, there are important functional implications of E-cad degradation. Though we have no direct in vivo evidence that degradation of E-cad by NE leads to lung epithelial disruption and injury, our findings still suggest strongly that this protease contributes to this physiopathologic phenotype. Because the N-terminal region corresponds to E-cad homophilic interaction hence cell-cell adhesion, its cleavage by NE (see below) is likely to disrupt and/or destabilize the epithelium integrity. This is consistent with our findings of epithelial monolayer disruption following its exposure to NE. It also implies that in the setting of lung injury, NE-mediated E-cad degradation likely participates to epithelial barrier alteration contributing to increased alveolocapillary membrane permeability and exudate within airspaces. While our in vivo finding with NE−/− mice show that NE deficiency was associated with considerable decrease of E-cad degradation, lung inflammation and injury of these mice was, however, similar to that seen in WT mice including inflammatory cell influx and albumin levels. Among the explanations could be that other proteases (e.g., CG and PR3) cleave E-cad masking therefore the relative contribution of NE. Some matrix metalloproteases (MMP) including MMP-7 cleave also E-cad [49, 50]. Although unlikely, the possibility that NE cleavage of E-cad corresponds to an epiphenomenon is plausible as well.

NE cleavage of E-cad was marked by the generation of a distinct fragment (about 80 kDa) in both cell culture system and in vivo. In their mouse model challenged with human NE, Evans SM and his colleagues reported the release into BAL fluids of a cleavage product with similar size and suggested NE as the prime protease targeting E-cad [25]. Given the estimated size of E-cad fragment detected by Western blotting and the epitope (amino acid sequence Asp157-Val709) that was used to raise anti-E-cad N-terminus antibody, we readily inferred that NE cleaves E-cad within its extracellular region. Furthermore, inspection of the primary structure of E-cad especially within the N-terminal domain (amino acid sequence ranging from 24 to 713, NCBI accession: Q9R0T4) revealed the presence of peptide bonds that are preferred by NE. Finally, Mayerle J. et al. identified a cleavage site for NE within EC-3 domain of E-cad extracellular portion (starting amino acid at position 394) [24]. Whether this cleaved fragment possesses in vivo biologic functions in inflamed lungs remains to be assessed. Interestingly, analysis of our immunostained lung tissue sections of infected mice suggested that E-cad fragments were engulfed by recruited neutrophils (left panel of Fig. 5b); an observation that confirms previously reported findings by Evans SM et al. [25]. It has been, however, shown in cell culture studies that cleaved E-cad fragment induces expression and activation of proteases such as matrix metalloproteases [51] suggesting that a hypothetical vicious cycle might exist between proteases and structural proteins (e.g., E-cad in this study), which perpetuates tissue inflammation and injury. Other studies reported that this E-cad fragment exhibits a stimulatory effect on the migratory capability of cells [52].

A common denominator of neutrophil-rich lung inflammatory diseases (e.g., ALI/ARDS, CF, non-CF bronchiectasis) is epithelial injury. Thus, cleavage of E-cad by neutrophil-derived NE, whose level and activity are known to be increased, could be anticipated. Such proteolytic event could occur during the migration of PMNs across the epithelium and/or their accumulation in the vicinity of epithelial lining. In this regard and as mentioned earlier, NE could degrade first other cell-cell junction proteins before E-cad (unpublished data, [27]. Also, the detected E-cad fragment in our mouse and human studies could include products derived from degraded VE-cad [28]; a hypothesis that warrants investigation. Other scenarios of NE involvement in E-cad degradation and ensued epithelium disruption could be envisioned. Recently, we reported that NE activates calpain, which is known to cleave E-cad [53, 54]. As mentioned above, tight junction and adherens junction proteins are spatially and functionally linked in epithelia. Degradation of E-cad could therefore lead to dysfunctionning of tight junctions and thus enhanced disruption of the epithelium [55].

It must be emphasized that in this study, we focused on the time point 24 h post-infection because it corresponds to a sharp increase of neutrophil numbers and enhanced NE activity by comparison to the other time points [37]. But, E-cad degradation could be seen at any time as long as significant amounts of free active NE are available and the microenvironment allows interaction of the enzyme and its target. As mentioned above, other proteases namely MMP have been reported to cleave E-cad [49, 50]. For example, McGuire JK et al. reported that MMP-7 mediates E-cadherin ectodomain shedding in injured lung epithelium [49, 50]. Interestingly, this group proposed that shedding of E-cad is required for epithelial repair. Altogether, previously reported findings along with this work point to E-cad cleavage as “double-edged sword” process and raise a relevant question of whether such cleavage contributes to dysfunctional or normal repair of injured epithelium. Our cell-free and cell culture data show that degradation of E-cad was blocked by NE physiological inhibitor, SLPI. As such, in vivo E-cad cleavage and associated phenotype (i.e., injury or repair) are in any case contingent on the equilibrium of active protease(s) (NE in this study) and their corresponding endogenous inhibitors within lung microenvironment.

## Conclusion

In conclusion, we provide in this study compelling evidence that NE contributes substantially to E-cad degradation in inflamed lung situations. However, based on our in vivo studies with mice deficient in NE, it seems that inhibition of this protease alone may not prevent proteolysis-mediated inflammation and tissue degradation seen in neutrophil-rich pathologies.

## Additional files

Additional file 1: Degradation of E-cad by NE, PR3 and CG. MLE15 cell protein extracts containing E-cadherin (10 μg) were incubated for 30 min alone (Ctrl) or in the presence of 25 nM of purified NE, PR3, and CG. The reactions were resolved by SDS-PAGE under reducing conditions and visualized by immunoblotting as in Fig. 1. Note that NE is the most potent protease to cleave E-cad followed by PR3 and CG. Molecular weight (kDa) standards are on the right. The findings are illustrative of at least three independent experiments. (TIFF 2703 kb)
Additional file 2: NE degrades cell-associated E-Cad. Confluent 16HBE cells were left untreated or treated with varying concentrations of purified NE (0, 2, 20, or 200 nM) for 6 h. Next, equal protein aliquots from cell lysates (10 μg) were subjected to SDS-PAGE and immunoblotting using antibodies raised against C-terminal parts of E-cad. A, Left panel. Anti-E-cad C-terminal antibody revealed a progressive decrease of E-cad that paralleled the increase of NE concentration. Right panel, densitometric analysis confirms decreased levels of E-cad. Data are mean values ± SD. **p* < 0.05; Kruskall-Wallis test. Of note, anti-E-cad C-terminal antibody detected varying fragments. Immunoblotting for GAPDH, an internal control, was used as protein loading control of cell lysate proteins. Experiments were repeated three times. (TIFF 2703 kb)
Additional file 7: Cell influx, albumin levels, and LDH release in BAL fluids of NE^−/−^ and WT mice in response to *P. aeruginosa* infection 24 h post-challenge. A. Total leukocyte counts in BAL fluids from NE^−/−^ and WT mice (*n* = 4/genotype) following i.n. challenge with a sub-lethal dose of *P. aeruginosa*. B. Representative cytospin micrographs of BAL fluids from both WT and NE−/− mice. Left panel, insignificant cell numbers corresponding mostly to resident alveolar macrophages, were detected in BAL fluids from saline control mice. Right panel, predominance of neutrophils. Scale bar, 50 μm. C. Albumin levels in cell-free BAL fluids from mice in A. D. LDH release in cell-free BAL fluids from mice in A. Data are mean values ± SD. **p* < 0.05; differences between genotypes of mice were tested by two-way analyses of variances with group and time as factors. (TIFF 2703 kb)

## Abbreviations

ALI: Acute lung injury
ARDS: Acute respiratory distress syndrome
BAL: Bronchoalveolar lavage
CF: Cystic fibrosis
CG: Cathepsin G
COPD: Chronic obstructive pulmonary diseases
E-cad: Epithelial-cadherin
fMLP: formyl-methionyl-leucyl-phenylalanine
i.n.: intranasal
LDH: Lactate dehydrogenase
MMP: Matrix metalloproteases
NE: Neutrophil elastase
NE−/−: NE-deficient mice
PMNs: Polymorphonuclear neutrophils
PR3: proteinase 3
SDS-PAGE: Sodium-dodecyl-sulfate polyacrylamide gel electrophoresis
SLPI: Secretory leukocyte proteinase inhibitor
WT: Wild type
