# Supplementary figures and images for: Neutrophil elastase cleaves epithelial cadherin in acutely injured lung epithelium
Source: Respir Res. 2016 Oct 17;17:129. doi: 10.1186/s12931-016-0449-x (PMC5067913; doi:10.1186/s12931-016-0449-x)

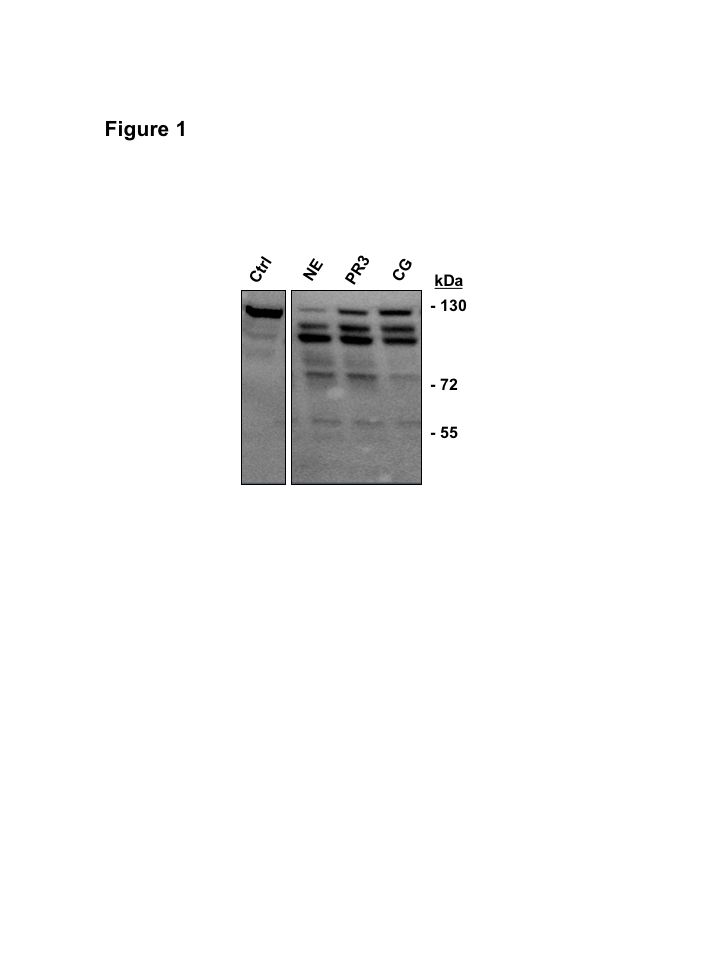

Supplement: Additional file 1: — Degradation of E-cad by NE, PR3 and CG. MLE15 cell protein extracts containing E-cadherin (10 μg) were incubated for 30 min alone (Ctrl) or in the presence of 25 nM of purified NE, PR3, and CG. The reactions were resolved by SDS-PAGE under reducing conditions and visualized by immunoblotting as in Fig. 1. Note that NE is the most potent protease to cleave E-cad followed by PR3 and CG. Molecular weight (kDa) standards are on the right. The findings are illustrative of at least three independent experiments. (TIFF 2703 kb) [file 12931_2016_449_MOESM1_ESM.tiff]

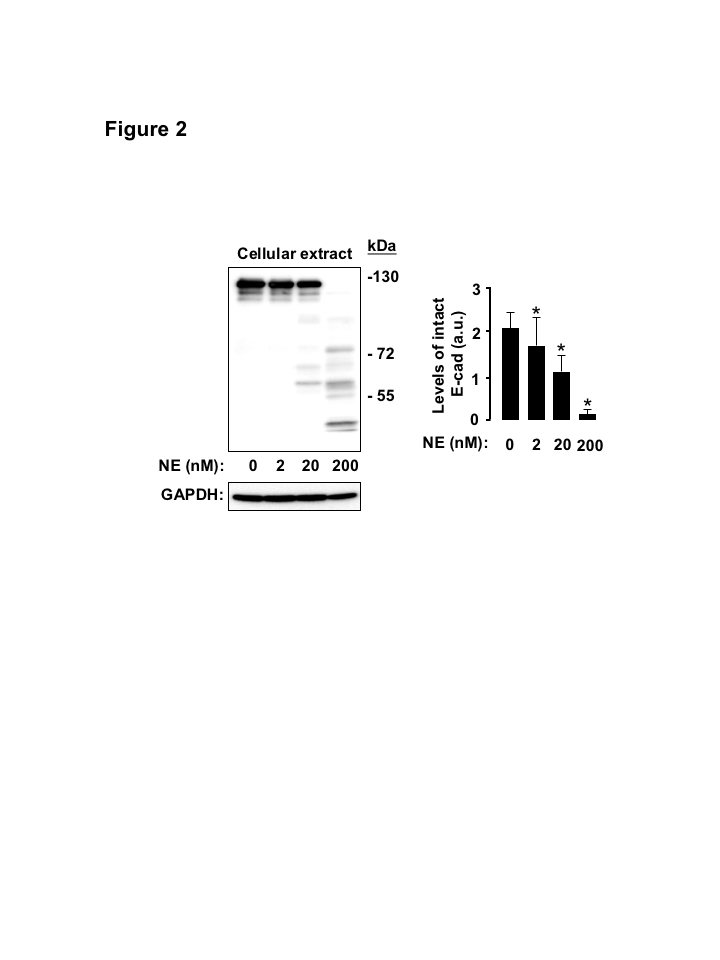

Supplement: Additional file 2: — NE degrades cell-associated E-Cad. Confluent 16HBE cells were left untreated or treated with varying concentrations of purified NE (0, 2, 20, or 200 nM) for 6 h. Next, equal protein aliquots from cell lysates (10 μg) were subjected to SDS-PAGE and immunoblotting using antibodies raised against C-terminal parts of E-cad. A, Left panel. Anti-E-cad C-terminal antibody revealed a progressive decrease of E-cad that paralleled the increase of NE concentration. Right panel, densitometric analysis confirms decreased levels of E-cad. Data are mean values ± SD. *p < 0.05; Kruskall-Wallis test. Of note, anti-E-cad C-terminal antibody detected varying fragments. Immunoblotting for GAPDH, an internal control, was used as protein loading control of cell lysate proteins. Experiments were repeated three times. (TIFF 2703 kb) [file 12931_2016_449_MOESM2_ESM.tiff]

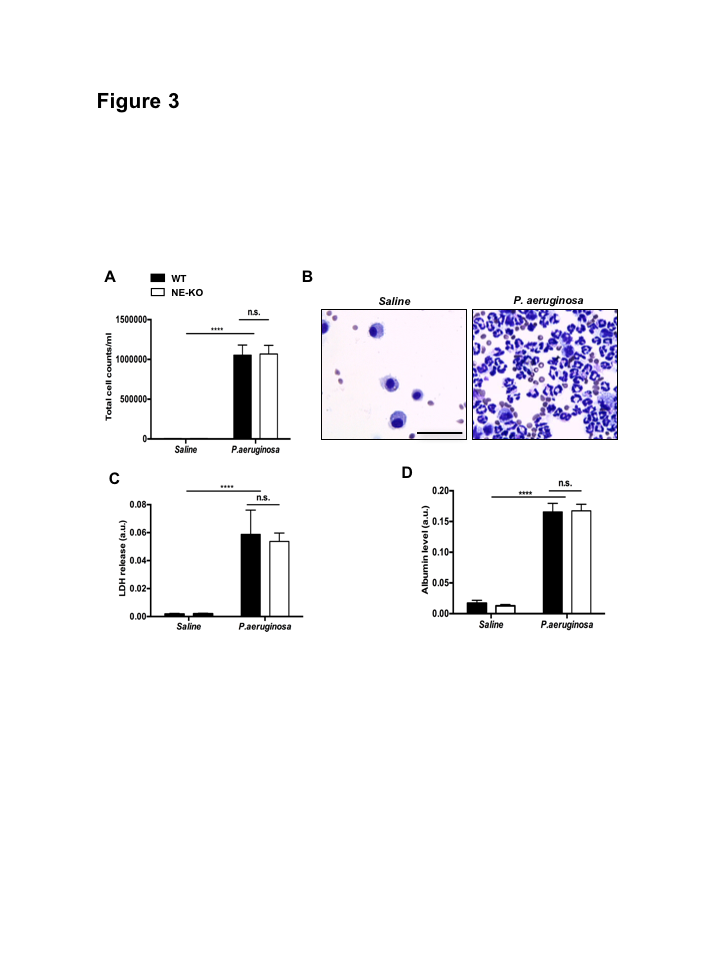

Supplement: Additional file 7: — Cell influx, albumin levels, and LDH release in BAL fluids of NE−/− and WT mice in response to P. aeruginosa infection 24 h post-challenge. A. Total leukocyte counts in BAL fluids from NE−/− and WT mice (n = 4/genotype) following i.n. challenge with a sub-lethal dose of P. aeruginosa. B. Representative cytospin micrographs of BAL fluids from both WT and NE−/− mice. Left panel, insignificant cell numbers corresponding mostly to resident alveolar macrophages, were detected in BAL fluids from saline control mice. Right panel, predominance of neutrophils. Scale bar, 50 μm. C. Albumin levels in cell-free BAL fluids from mice in A. D. LDH release in cell-free BAL fluids from mice in A. Data are mean values ± SD. *p < 0.05; differences between genotypes of mice were tested by two-way analyses of variances with group and time as factors. (TIFF 2703 kb) [file 12931_2016_449_MOESM7_ESM.tiff]
